# Normal Values for Echocardiographic Myocardial Work in a Large Pediatric Population

**DOI:** 10.3390/diagnostics14101022

**Published:** 2024-05-15

**Authors:** Pietro Marchese, Marco Scalese, Nadia Assanta, Eliana Franchi, Cecilia Viacava, Giuseppe Santoro, Giulia Corana, Alessandra Pizzuto, Francesca Valeria Contini, Shelby Kutty, Massimiliano Cantinotti

**Affiliations:** 1 Fondazione G. Monasterio CNR-Regione Toscana, 54100 Pisa, Italy; pmarchese@ftgm.it (P.M.); assanta@ftgm.it (N.A.); franchi@ftgm.it (E.F.); cecilia.viacava@ftgm.it (C.V.); gsantoro@ftgm.it (G.S.); corana@ftgm.it (G.C.); apizzuto@ftgm.it (A.P.); 2 Istituto di Scienze Della Vita (ISV), Scuola Superiore Sant’Anna, 56127 Pisa, Italy; 3 Department of Statistics, National Research Institute, CNR, 56124 Pisa, Italy; scalese@ifc.cnr.it; 4 Clinical Cardiology Unit, Cagliari University, 09042 Monserrato, Italy; fvcontini@gmail.com; 5 Taussig Heart Center, Department of Pediatrics, Johns Hopkins Hospital, Baltimore, MD 21204, USA; skutty1@jhmi.edu

**Keywords:** myocardial work, normal values, echocardiography, children

## Abstract

Background: Echocardiographic myocardial work is a new load-independent echocardiographic technique to quantify left ventricle (LV) systolic performance. Our aim was to establish normal values for echocardiographic myocardial work in a large population of healthy children. Methods: For all the subjects 4-, 2-, and 3-chamber-view videos were stored. The following parameters were obtained by offline analysis: the global myocardial work (GMW), the global myocardial constructive work (GCW), the global myocardial wasted work (GWW), and the global myocardial work efficiency (GWE). Age, weight, height, heart rate, and body surface area (BSA) were used as independent variables in the statistical analysis. Results: In all, 516 healthy subjects (age range, 1 day—18 years; median age, 8.2 ± 5.3 years; 55.8% male; body surface area (BSA) range, 0.16 to 2.12 m^2^) were included. GWI, GCW, and GWW increased with weight, height, and BSA (ρ ranging from 0.635 to 0.226, *p* all < 0.01); GWI and GCW positively correlated with age (ρ 0.653 and 0.507). After adjusting for BSA differences, females showed higher mean GWI (*p* = 0.002) and GCW values (*p* < 0.001), thus Z-score equations for gender have been presented. Conclusions: We provided MW values in a large population of healthy pediatric subjects including lower ages. MW values increased with age and body size and, interestingly, were higher in females than in men. These data cover a gap in current nomograms and may serve as a baseline for the evaluation of MW analysis in children with congenital and acquired heart diseases.

## 1. Background

Myocardial work (MW) assesses left ventricular (LV) systolic performance independent of load that affects conventional ejection fraction and speckle tracking echocardiography (STE) strain analysis [1,2]. Both pressure and strain vary with time through the cardiac cycle. One can be plotted against the other, and a pressure–strain loop emerges for the cardiac cycle beginning as the mitral valve opens and the ventricle fills, proceeding through isovolumic contraction, ventricular ejection, and finally isovolumic relaxation [1,2]. Myocardial work calculations incorporate the area of the pressure–strain loop during LV ejection [1,2,3,4,5]. Dynamic pressure–strain loops can be created from echocardiographic measurements of myocardial strain in three apical views and a noninvasive cuff blood pressure measurement performed at the same time. The timing of the aortic and mitral valves opening and closing are needed to transform the data and plot the loop [1,2]. There has been considerable progress made in the software available to convert images and pressure into a rendering that is useful for analyzing myocardial work [1,2]. Power is the rate of doing work; when power is graphed against time, four myocardial work parameters can be distinguished [1,2]. The global MW index is the total work within the area of the pressure–strain loop during left ventricle ejection [1,2]. Constructive work (CW) is work performed during segmental shortening and reversed during isovolumetric relaxation. Wasted work (WW) is work performed during segmental elongation and reversed during isovolumetric relaxation. Finally, work efficiency is the fraction of constructive work divided by constructive work plus wasted work [1,2,3,4,5,6]. Myocardial strain–LV loop analysis furthermore allows for differentiation between constructive and wasted work and provides data on energy consumption [1,2,3,4,5]. Adult studies showed how MW may provide additional and complementary information for the analysis of LV function compared to myocardial strain which is influenced by the afterload [6]. Normal values for MW have been derived in adults [6,7,8,9,10,11]. The few studies on normal MW parameters in children [1,2,3,4,5] used limited sample size (150 to 212 subjects) and mostly evaluated children above 6 years [3,4,5]. Data on younger children are limited [2], and data in neonates and infants are absent.

Normal reference standards for each of the myocardial work parameters would be required to accurately interpret them in the pediatric setting. Our aim was to establish normal values for echocardiographic myocardial work parameters in a large population of healthy children across the pediatric spectrum.

## 2. Methods

Healthy Caucasian children were prospectively recruited at a single center from July 2023 to December 2023. This study was approved by the local ethics committee (Study ‘‘Bet’’ No. 390). Parents, tutors, or legal guardians were informed of the aims and significance of this study, and they all agreed to participate by providing written consent. Parents, tutors, or legal guardians were informed that no sedation was performed, and no adjunctive examinations were performed excluding those routinely performed during outpatient care. Participation in this study only indicated permission for off-analysis on echocardiographic images routinely stored and anonymized statical analysis. Exclusion criteria have been previously reported [12,13]. All subjects with clinical, electrocardiographic, or echocardiographic evidence of congenital or acquired heart disease were excluded. Other exclusion criteria consisted of patients with known or suspected neuro-muscular disease, genetic syndromes, or chromosomal abnormalities. Obese children with a body mass index (BMI) >95th percentile for children <2 years old [12,13] or weight-for-length z-score >2 based on the World Health Organization (WHO) Child Growth Standards for children <2 years old [12,13] were also excluded. Other criteria of exclusion were pulmonary hypertension; systemic hypertension (for children >4 years of age); connective tissue disease; or a family history of genetic cardiac disease (such as Marfan syndrome or cardiomyopathy) [12,13]. Children with significant abnormalities of chest wall (e.g., pectus excavatum, keeled chest, etc.) were also excluded. All non-Caucasian subjects were excluded to avoid racial variability bias.

### 2.1. Echocardiographic Measurements and Offline Analysis

All patients underwent a complete color flow Doppler and tissue Doppler examination, and images were digitally stored for subsequent offline analysis. Subjects were not sedated, and thus, only images obtained by cooperative, quiet children were collected for the final analysis.

**Figure 1:**
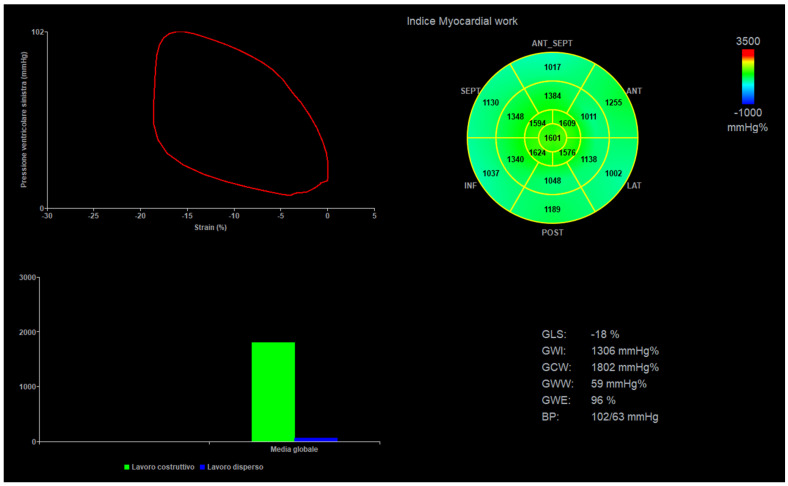
Myocardial work curves, BP = blood pressure, GLS = global longitudinal strain, GWI = global myocardial work index, GWC = global myocardial constructive work, GWW = global myocardial wasted work, and GWE = global myocardial work efficiency (GWE).

Acquisition of 4-, 2-, and 3-chamber views was performed at a frame rate of 60 to 120 frames/s, using the Vivid E95 system (General Electric, GE Healthcare, Chicago, IL, USA). Clips with at least 5 betas were stored. Two experienced pediatric cardiologists (M.C. and E.F.) acquired the images, and two other experienced operators (P.M. and N.A.) performed offline analysis on a dedicated workstation (Echopac V.202, General Electric, GE Healthcare, Chicago, IL, USA). Only good clips, with clear border contours and no phenomenon of out-plane were selected for the final analysis. Speckle tracking echocardiography analysis was performed in semiautomatic fashion with manual correction. Global LV longitudinal strain (LVGL) and regional (basal, mid, and apical) values were automatically derived from all views measured with a standard 6-segment model (e.g., 6 segments for each view, 18 segments globally) [1,2,3,4,5]. Left ventricular ejection fraction was calculated by the biplane Simpson’s method in a semi-automated fashion with manual correction. For the final analysis, single basal, mid, and apical segments values were summed, and a mean was obtained [14,15]. Left ventricle mass was calculated by left ventricle M-mode in a short axis view [14,15]. A mean of 3 values of noninvasive systolic and diastolic blood pressure—obtained by a brachial-cuff aneroid sphygmomanometer and taken during the imaging acquisition—were plugged into the software, and MW indexes were automatically generated. The following parameters were evaluated: global myocardial work index (GWI), global myocardial constructive work (GCW), global myocardial wasted work (GWW), and global myocardial work efficiency (GWE) (Figure 1).

### 2.2. Statistical Analysis

To examine the relationship among parameters of body size, heart rate, age, and each of the echocardiographic variables, multiple models using linear, logarithmic, exponential, and square root equations were tested. To test the goodness-of-fit of the model, we used the coefficient of determination (R^2^) that is expressed as a value between zero and one. A value of one indicates a perfect fit and, therefore, a very reliable model, while a value of zero would indicate that the model fails to accurately model the dataset. The fit was considered inadequate with a low R^2^ (i.e., below 0.5) [12,13]. The model with the highest adjusted coefficient of determination value was considered to provide the best fit. When the models were multivariable, adjusted R^2^ was used, which is a modification of R^2^ that adjusts for the number of terms in a model. Coefficient of determination always increases when a new term is added to a model, but adjusted R^2^ increases only if the new term improves the model more than would be expected by chance [12,13]. The presence or absence of heteroscedasticity, a statistical term used to describe the behavior of variance and normality of the residuals, was also tested by the White test and the Breusch–Pagan test as described previously [12,13]. The Shapiro–Wilk test was used to assess the normality of distribution [12,13]. First, age, weight, height, heart rate (HR), and body surface area (BSA) [12,13] were used as the independent variables in different univariate regression analyses to predict the mean values of each echocardiographic measurement. Second, the variables that maintained a statistical significance of 0.1 were included into the stepwise multivariable procedures and a *p*-value < 0.05 was considered significant in the final model. The Haycock formula was used to calculate BSA [12,13]. The effects of confounding factors such as gender, prematurity, and type of delivery were also evaluated, as previously described [12,13]. Z-scores (a standardized value that indicates by how many standard deviations a value is above or below the mean in a normally distributed population) are commonly used for normalization in pediatric echocardiography. Z-scores are computed by dividing the residual values by the modeled standard errors of residual values [12,13]. The presence of a high coefficient of determination [12,13] is essential to compute z-scores with sufficient statistical power.

Rates of intra-observer and inter-observer variability were calculated from 20 randomly selected subjects. The sample size needed to obtain nomograms with sufficient statistical power was calculated as previously described [12,13] by dividing the population into 6 major age stages (Group 1, neonates: 0–30 days; Group 2, infancy: 31 days-12 months; Group 3, toddlers: 13 months-2 years; Group 4, early childhood: 2–5 years; Group 5, middle childhood: 6–11 years; Group 6, early adolescence: 12–17 years).

Statistical Package for the Social Sciences (SPSS) Release 13.0 (Chicago, IL, USA) and Stata Version 10 for Windows (Stata Corp, College Station, TX, USA, 2001) were used for analyses.

## 3. Results

From the 550 patients enrolled, 34 were excluded for poor image quality or incomplete acquisition. Accordingly, the feasibility was 93.81%. The final population included 516 healthy subjects (range, 1 days-18 years; median age 8.2 ± 5.3 years; 55.8% male), and BSA from 0.16 to 2.12 m^2^. See Table 1 and Table 2.

### 3.1. Correlation of Myocardial Work Parameters

GWI, GCW, and GWW positively correlated with weight, height, BSA, systolic, and diastolic blood pressure (ρ ranging from 0.635 to 0.226, *p* all < 0.01). GWI and GCW positively correlated with age (ρ 0.653 and 0.507) and left ventricle mass (rho = 0.567, *p* < 0.001), GCW (rho = 0.445, *p* < 0.001), and negatively with HR (ρ from 0.732 to 0.130, *p* all < 0.01). All MW parameters correlated with one another (ρ from 0.809 to 0.110, most of the *p* < 0.01) and with left ventricle global longitudinal strain (GLS) (ρ from 0.470 to 0.240, *p* all < 0.01). Correlations of MW parameters with left ventricle ejection fraction (EF) were insignificant (ρ from 0.074 to 0.03, *p* all > 0.05). (Supplementary Table S1).

### 3.2. Building of Z-Scores

**Figure 2:**
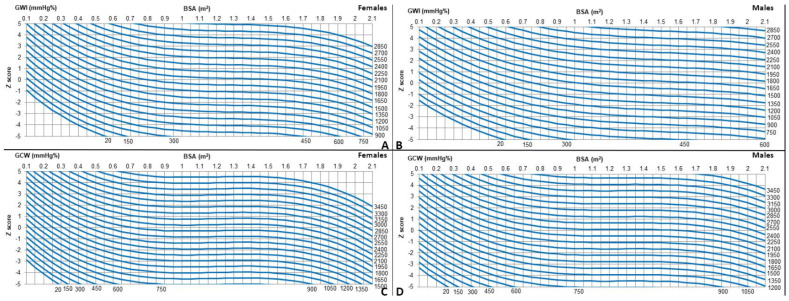
(**A**–**D**) Z-scores charts for myocardial work indices by gender.

The measurements were first modeled with HR, age, weight, height, and BSA. BSA provided the best fit. For all measurements, linear, logarithmic, exponential, and square root models were evaluated for best fit, and tests for heteroscedasticity were applied. The best-fit models, satisfying the assumption of homoscedasticity and normality of residuals and showing the highest coefficient of regression (R^2^) score, were cubic (y = a + b_1_*x + b_2_*x^2^ + b_3_*x^3^) (Table 3 and Table 4). Z-scores for the whole population are provided in Table 5 and Figure 2. Z-scores are provided only for GWI and GCW, since for GWW and GWE, R^2^ values were too low to derive Z-scores equations with sufficient statistical power.

### 3.3. Confounders: Gender

Regression models incorporating gender as a covariate, after adjusting for BSA differences, showed females having higher mean GWI (*p* = 0.002) and GCW values (*p* < 0.001). Therefore, different Z-score equations for males and females have been generated (Table 2 and Figure 1). The inter-observer and intra-observer coefficients showed good reproducibility (Supplementary Table S2).

## 4. Discussion

We report echocardiographic myocardial work values in a large population of healthy Caucasian children of different ages. Compared to previous reports [1,2,3,4,5], not only did we use a larger sample size to derive normal values, but we also had the full pediatric age spectrum, including lower ages. While previous studies found no association of myocardial work parameters with age (2.4) and body size (2.5), we found a significant increase in myocardial work values with increasing age and somatic growth.

Only a previous pediatric series [3] of 183 healthy children (93 males; 6–13 years) revealed a similar positive correlation of myocardial work values with age and somatic growth; global work index values positively correlated with age, height, weight, and body surface area (β coefficient of 0.63, 0.61, 0.61, and 0.64, respectively, all *p* < 0.001); on the other hand, global constructive work strongly correlated with body surface area (β coefficient 0.51, *p* < 0.001) and moderately with age, height, and weight (β coefficient 0.48, 0.48, and 0.50, respectively, all *p* < 0.001) [3].

We also found the association of echocardiographic-derived myocardial work values with gender, that relatively surprisingly revealed higher values in females. The data we presented are different from previous pediatric observations showing either no gender influence [2,4,5,6], or slightly higher values in males [3]. Our data deriving from a larger and uniform population are, however, in line with those of the literature on adults [7,8,9,10]. Adult studies, in fact, revealed slightly higher global work indices [7,8,10,11], global constructive work values, and global waste work values [11], and lower global wasted work values in women [10]. The trend of growth with age and somatic growth slightly differs among different parameters of myocardial work response. For the global work index, the increase in values is quite homogenous. To have an idea of the degree of increase in myocardial work values with increasing age and somatic growth, we may take the example of global work index values, where mean values varied from 559.5 mmHg% in a female neonate with a BSA of 0.2. to 1580 mmHg% in a female adolescent with a BSA >1.0. However, values of global work indices in adolescence on average remain lower than those in adults [8,9,10,11], except for high body surface areas. For instance, the global work index reaches values of 1800–1900 mmHg% (corresponding to mean adult values, e.g., 1920 mmHg%) [9] only from a BSA of 1.9 m^2^. Global constructive work values instead increased more rapidly with age and body size, reaching ranges of normality like those in adults (e.g., 2200 mmHg%) at a BSA of 1.6 m^2^ in females. Global constructive work values in men also increased rapidly but reached values like those in adults only for large body sizes (e.g., BSA from 1.9 m^2^).

Our data reflect previous observations [2,3,4,5,6], showing significant statistical associations between both the global work index and global constructive work with systolic blood pressure [2,3,4,5,6] (β coefficient = 0.444–0.878, *p* < 0.001) and left ventricle global longitudinal strain [2,3,4,5,6] (β coefficient = 0.233–0.796, *p* < 0.001–0.003). This is, however, not surprising since myocardial work values derive from the pressure–strain loop [1,2]. More interestingly, as previously described [5,6], we confirm a weak correlation of myocardial work parameters with left ventricle ejection fraction (by Simpson’s method). This underscores the differences of parameters deriving from speckle tracking echocardiography (including myocardial work parameters) from classical left ventricular ejection fraction in terms of left ventricle systolic function assessment [16,17,18]. New data deriving from myocardial work analysis may augment the utility and the diagnostic accuracy of classical speckle tracking echocardiography parameters (e.g., global longitudinal strain), to underscore subtle left ventricular myocardial dysfunction (not visible with conventional left ventricular ejection fraction) and to monitor even minimal variation during the follow-up [14,15,16].

## 5. Strengths and Limitations

This study has some strengths. The nomograms we generated are based on a large homogenous cohort published so far, with children of all ages, including lower ages (whose data were extremely limited). This study has also a few limitations. We evaluated only the Caucasian ethnic group. However, this eliminated bias due to differing racial compositions and will allow future comparisons with populations of different ethnicities [13]. Other studies, including different ethnic groups, are advised to evaluate the potential influence of ethnicity on myocardial work parameters in children [12,13]. We did not evaluate the potentially strong correlation between MW indices and the children’s chest wall conformation [18,19]. Children with significant pectus excavatum or other chest wall deformities, that may have significantly altered myocardial strain parameters [19], however, were excluded for the present investigation. The software used was vendor-specific. At present, however, there are limited vendors offering this analysis, thus a comparison among different software is not feasible yet. MW estimates incorporate strain and all the inherent limitations of deformation imaging. The increases in end-diastolic volume and stroke volume do not translate directly and linearly into an increase in strain. Finally, maintaining wall stress represents ‘internal’ work the ventricle must do but is not included in the myocardial work measures, where the area of the pressure–strain curve describes ‘external’ work.

### Conclusive Remarks

In conclusion, we report echocardiographic myocardial work values in a large population of healthy children of different ages. These data may serve as a baseline for the evaluation of MW analysis in children with congenital and acquired heart disease.

## Figures and Tables

**Table 1:** Demographic data.

	Mean	SD	Median	Percentile 25	Percentile 75	Minimum	Maximum
Weight (kg)	31.8	20.0	28.0	16.0	47.0	2.1	85.0
Height (cm)	124.7	37.2	130.0	103.0	155.0	39.0	190.0
HR (bpm)	95.1	27.8	87.0	76.0	108.0	50.0	183.0
Age (years)	8.2	5.3	8.4	3.9	12.4	0.0	18.0
BSA (m^2^)	1.03	0.48	1.00	0.67	1.42	0.16	2.12

HR = heart rate, BSA = body surface area, SD = standard deviation.

**Table 2:** Distribution by age groups.

Age	N	%
0–30 days	25	4.8
1–12 months	49	9.5
1–2 years	20	3.9
2–5 years	64	12.4
5–11 years	175	33.9
11–17 years	183	35.5
Total	516	100.0

**Table 3:** Coefficients for regression equations relating echocardiographic measurements and body surface area, the standard error of the estimate, and the determination coefficient. Normality test: Shapiro–Wilk and Lilliefors (Kolmogorov–Smirnov). Heteroscedasticity test (White test and Breusch–Pagan test). BSA HAYCOCK. (y = a + b1*x + b2*x2 + b3*x3); Z value = (Measurement –(a + b1*x + b2*x2 + b3*x3))/√MSE.

Measurement	a	b_1_	b_2_	b_3_	SEE (√MSE)	R^2^	SW	KS	BP	W
GWI (mmHg%)	56.8	3054.0	−2045.8	471.5	244.51	0.601	0.202	0.200	0.002	0.169
GCW (mmHg%)	526.3	3883.6	−3086.4	806.1	276.67	0.501	0.120	0.200	0.097	0.219

**Table 4:** Coefficients for regression equations relating echocardiographic measurements and body surface area, the standard error of the estimate, and the determination coefficient. Normality test: Shapiro–Wilk and Lilliefors (Kolmogorov–Smirnov). Heteroscedasticity test (White test and Breusch–Pagan test). BSA HAYCOCK. (y = a + b1*x + b2*x2 + b3*x3); Z value = (Measurement –(a + b1*x + b2*x2 + b3*x3))/√MS..E.

Measurement	a	b_1_	b_2_	b_3_	SEE (√MSE)	R^2^	SW	KS	BP	W
Male										
GWI (mmHg%)	136.5	2637.6	−1640.3	358.5	235.0	0.620	0.159	0.063	0.003	0.226
GCW (mmHg%)	552.9	3643	−2883	756.2	262.2	0.514	0.717	0.678	0.045	0.261
Female										
GWI (mmHg%)	−108.7	3910.8	−3006.2	784.1	250.8	0.598	0.899	0.640	0.173	0.068
GCW (mmHg%)	404.0	4642.4	−3969.1	1109.2	283.0	0.507	0.731	0.421	0.866	0.087

BP, Breusch–Pagan test; KS, Kolmogorov–Smirnov test; SEE, standard error of the estimate; SW, Shapiro–Wilk test; W, White test. GWI, global myocardial work index; GCW, global myocardial constructive work.

**Table 5:** Predicted values (mean ± 2SD) of measured echocardiography variables expressed by body surface area (BSA) (Haycock), FEMALE.

FEMALES											
	0.20	0.25	0.30	0.35	0.40	0.50	0.60	0.70	0.8	0.9	1.0
	57.9	191.8	313.6	423.9	523.2	691.6	823.4	923.2	995.9	1046.1	1078.5
GWI	559.5	693.4	815.2	925.4	1024.8	1193.2	1324.9	1424.8	1497.5	1547.7	1580.1
	1061.1	1194.9	1316.7	1427.0	1526.4	1694.8	1826.5	1926.4	1999.1	2049.3	2081.7
	616.6	767.8	903.4	1024.2	1130.9	1305.6	1434.1	1523.3	1579.6	1609.8	1620.5
GCW	1182.6	1333.8	1469.4	1590.2	1696.9	1871.6	2000.1	2089.3	2145.6	2175.8	2186.5
	1748.6	1899.8	2035.4	2156.2	2262.9	2437.6	2566.1	2655.3	2711.6	2741.8	2752.5
	1.1	1.2	1.3	1.4	1.5	1.6	1.7	1.8	1.9	2.0	2.1
	1097.9	1108.8	1116.1	1124.5	1138.6	1163.1	1202.8	1262.4	1346.5	1459.9	1607.2
GWI	1599.4	1610.4	1617.7	1626.1	1640.1	1664.7	1704.4	1763.9	1848.1	1961.5	2108.8
	2101.0	2112.0	2119.3	2127.6	2141.7	2166.3	2206.0	2265.5	2349.6	2463.0	2610.4
	1618.4	1610.1	1602.2	1601.6	1614.7	1648.2	1708.9	1803.3	1938.1	2120.0	2355.6
GCW	2184.4	2176.1	2168.2	2167.6	2180.7	2214.2	2274.9	2369.3	2504.1	2686.0	2921.6
	2750.4	2742.1	2734.2	2733.6	2746.7	2780.2	2840.9	2935.3	3070.1	3252.0	3487.6
MALES											
	0.20	0.25	0.30	0.35	0.40	0.50	0.60	0.70	0.8	0.9	1.0
	131.2	228.9	319.8	404.0	482.0	620.0	735.9	832.0	910.3	973.0	1022.2
GWI	601.3	699.0	789.9	874.1	952.1	1090.1	1206.0	1302.1	1380.4	1443.0	1492.3
	1071.4	1169.1	1259.9	1344.2	1422.1	1560.1	1676.1	1772.1	1850.4	1913.1	1962.4
	647.9	770.9	882.4	982.9	1072.9	1223.9	1339.9	1425.5	1485.2	1523.5	1545.0
GCW	1172.2	1295.3	1406.8	1507.3	1597.3	1748.3	1864.3	1949.9	2009.6	2047.9	2069.4
	1696.6	1819.7	1931.2	2031.6	2121.7	2272.7	2388.7	2474.3	2533.9	2572.3	2593.8
	1.1	1.2	1.3	1.4	1.5	1.6	1.7	1.8	1.9	2.0	2.1
	1060.2	1089.0	1110.8	1127.7	1142.0	1155.7	1171.0	1190.1	1215.1	1248.2	1291.5
GWI	1530.2	1559.0	1580.8	1597.8	1612.1	1625.8	1641.1	1660.2	1685.2	1718.3	1761.5
	2000.3	2029.1	2050.9	2067.9	2082.2	2095.9	2111.2	2130.3	2155.3	2188.3	2231.6
	1554.2	1555.7	1553.9	1553.5	1559.0	1574.8	1605.6	1655.8	1730.1	1832.9	1968.8
GCW	2078.6	2080.1	2078.3	2077.9	2083.3	2099.2	2130.0	2180.2	2254.5	2357.3	2493.2
	2603.0	2604.4	2602.7	2602.3	2607.7	2623.6	2654.3	2704.6	2778.9	2881.7	3017.6

The estimated values are in bold, the values above are −2SD, and the values below are +2SD. GWI = global myocardial work index, GCW = global myocardial constructive work.

## Data Availability

The data presented in this study are available on request from the corresponding author. The data are not publicly available due to privacy issue.

## Supplementary Materials

The following supporting information can be downloaded at: https://www.mdpi.com/article/10.3390/diagnostics14101022/s1, Table S1: Spearman’s rho correlation coefficient; Table S2: Inter- and intra-observer reliability analysis.

## Abbreviations

BSA	body surface area,
BP	Breusch–Pagan test,
CV	coefficient of variation,
DAp	diastolic arterial pressure,
EF	ejection fraction,
HR	heart rate,
ICC	intraclass correlation coefficient,
KS	Kolmogorov–Smirnov test,
LV	left ventricle,
MW	myocardial work,
GWI	global myocardial work index,
GCW	global myocardial constructive work,
GWW	global myocardial wasted work,
GWE	global myocardial work efficiency,
GLS	global longitudinal strain,
SAP	systolic arterial pressure,
SEE	standard error of the estimate,
STE	speckle tracking echocardiography,
SW	Shapiro–Wilk test,
W	White test.

## References

[1] Sabatino J., Borrelli N., Fraisse A., Herberg J., Karagadova E., Avesani M., Bucciarelli V., Josen M., Paredes J., Piccinelli E. (2021). Abnormal myocardial work in children with Kawasaki disease. Sci. Rep..

[2] Sabatino J., Leo I., Strangio A., Bella S., Borrelli N., Avesani M., Josen M., Paredes J., Piccinelli E., Sirico D. (2022). Echocardiographic Normal Reference Ranges for Non-invasive Myocardial Work Parameters in Pediatric Age: Results From an International Multi-Center Study. Front. Cardiovasc. Med..

[3] Cui C., Zheng Q., Li Y., Huang D., Hu Y., Wang Y., Liu R., Liu L., Zhang L. (2022). Reference Values of Noninvasive Myocardial Work Indices Measured by Echocardiography in Healthy Children. Front. Pediatr..

[4] Pham T.T.M., Truong V.T., Vu P.N., Tran T.X., Nguyen N.N.H., Nguyen L.P.T., Tu H.N.T., Palmer C., Tretter J.T., Levy P. (2022). Echocardiographic Reference Ranges of Non-invasive Myocardial Work Indices in Children. Pediatr. Cardiol..

[5] Luo X., Ge Q., Su J., Zhou N., Li P., Xiao X., Chen Y., Wang D., Ma Y., Ma L. (2022). Normal ranges of non-invasive left ventricular myocardial work indices in healthy young people. Front. Pediatr..

[6] Olsen F.J., Skaarup K.G., Lassen M.C.H., Johansen N.D., Sengeløv M., Jensen G.B., Schnohr P., Marott J.L., Søgaard P., Gislason G. (2022). Normal Values for Myocardial Work Indices Derived from Pressure-Strain Loop Analyses: From the CCHS. Circ. Cardiovasc. Imaging.

[7] Manganaro R., Marchetta S., Dulgheru R., Ilardi F., Sugimoto T., Robinet S., Cimino S., Go Y.Y., Bernard A., Kacharava G. (2019). Echocardiographic reference ranges for normal non-invasive myocardial work indices: Results from the EACVI NORRE study. Eur. Heart J. Cardiovasc. Imaging.

[8] Morbach C., Sahiti F., Tiffe T., Cejka V., Eichner F.A., Gelbrich G., Heuschmann P.U., Störk S., STAAB consortium (2020). Myocardial work—Correlation patterns and reference values from the population-based STAAB cohort study. PLoS ONE.

[9] Truong V.T., Vo H.Q., Ngo T.N., Mazur J., Nguyen T.T., Pham T.T., Le T.K., Phan H., Palmer C., Nagueh S.F. (2022). Normal Ranges of Global Left Ventricular Myocardial Work Indices in Adults: A Meta-Analysis. J. Am. Soc. Echocardiogr..

[10] Jasaityte R., Bajraktarevic R., Blaschke-Waluga D., Seeland U., Regitz-Zagrosek V., Landmesser U., Stangl K., Knebel F., Stangl V., Brand A. (2023). Determinants of myocardial work indices in women. Echocardiography.

[11] Lindseth K.T., Gerdts E., Midtbø H., Pristaj N., Cramariuc D., Einarsen E. (2023). Myocardial Work in Middle-Aged Adults with Overweight and Obesity: Associations with Sex and Central Arterial Stiffness. J. Clin. Med..

[12] Cantinotti M., Scalese M., Giordano R., Franchi E., Assanta N., Marotta M., Viacava C., Molinaro S., Iervasi G., Santoro G. (2018). Normative Data for Left and Right Ventricular Systolic Strain in Healthy Caucasian Italian Children by Two-Dimensional Speckle-Tracking Echocardiography. J. Am. Soc. Echocardiogr..

[13] Cantinotti M., Kutty S., Franchi E., Paterni M., Scalese M., Iervasi G., Koestenberger M. (2017). Pediatric echocardiographic nomograms: What has been done and what still needs to be done. Trends Cardiovasc. Med..

[14] Lopez L., Saurers D.L., Barker P.C.A., Cohen M.S., Colan S.D., Dwyer J., Forsha D., Friedberg M.K., Lai W.W., Printz B.F. (2024). Guidelines for Performing a Comprehensive Pediatric Transthoracic Echocardiogram: Recommendations From the American Society of Echocardiography. J. Am. Soc. Echocardiogr..

[15] Lopez L., Colan S.D., Frommelt P.C., Ensing G.J., Kendall K., Younoszai A.K., Lai W.W., Geva T. (2010). Recommendations for quantification methods during the performance of a pediatric echocardiogram: A report from the Pediatric Measurements Writing Group of the American Society of Echocardiography Pediatric and Congenital Heart Disease Council. J. Am. Soc. Echocardiogr..

[16] Lin Y., Zhang L., Hu X., Gao L., Ji M., He Q., Xie M., Li Y. (2023). Clinical Usefulness of Speckle-Tracking Echocardiography in Patients with Heart Failure with Preserved Ejection Fraction. Diagnostics.

[17] Cantinotti M., Marchese P., Scalese M., Medino P., Jani V., Franchi E., Vitali P., Santoro G., Viacava C., Assanta N. (2021). Left Ventricular Systolic Impairment after Pediatric Cardiac Surgery Assessed by STE Analysis. Healthcare.

[18] Li Y., Xie M., Wang X., Lu Q., Zhang L., Ren P. (2015). Impaired right and left ventricular function in asymptomatic children with repaired tetralogy of Fallot by two-dimensional speckle tracking echocardiography study. Echocardiography.

[19] Sonaglioni A., Nicolosi G.L., Braga M., Villa M.C., Migliori C., Lombardo M. (2021). Does chest wall conformation influence myocardial strain parameters in infants with pectus excavatum?. J. Clin. Ultrasound..

