# Supplementary material for: Normal Values for Echocardiographic Myocardial Work in a Large Pediatric Population
Source: Diagnostics (Basel). 2024 May 15;14(10):1022. doi: 10.3390/diagnostics14101022 (PMC11120304; doi:10.3390/diagnostics14101022)
Supplement: Supplementary file 1 [file diagnostics-14-01022-s001.zip › diagnostics-2985277-supplementary.pdf]

Supplementary Table S1. Spearman's rho correlation coefficient.

		GWI	GCW	GWW	GWE	Age	Weight	Height	BSA	SAP	DAP	HR (bpm)	LVEF	LV GLS (%)
GWI (mmHg%)	rho		0.790 **	0.110 *	0.261 **	0.653 **	0.634 **	0.629 **	0.635 **	0.732 **	0.494 **	−0.567 **	0.074	0.361 **
	p		0.000	0.013	0.000	0.000	0.000	0.000	0.000	0.000	0.000	0.000	0.095	0.000
GCW (mmHg%)	rho	0.790 **		0.315 **	0.136 **	0.507 **	0.496 **	0.488 **	0.497 **	0.728 **	0.494 **	−0.406 **	0.053	0.470 **
	p	0.000		0.000	0.002	0.000	0.000	0.000	0.000	0.000	0.000	0.000	0.236	0.000
GWW (mmHg%)	rho	0.110 *	0.315 **		−0.809 **	0.084	0.226 **	0.227 **	0.227 **	0.286 **	0.130 **	−0.177 **	−0.031	−0.067
	p	0.013	0.000		0.000	0.056	0.000	0.000	0.000	0.000	0.003	0.000	0.477	0.126
GWE (%)	rho	0.261 **	0.136 **	−0.809 **		−0.025	0.044	0.041	0.044	0.054	0.091 *	−0.060	0.066	0.240 **
	p	0.000	0.002	0.000		0.566	0.313	0.347	0.322	0.223	0.039	0.171	0.136	0.000

**. Correlation is significant at the 0.01 level (2-tailed). *. Correlation is significant at the 0.05 level (2-tailed). GWI = global myocardial work index, GCW = global myocardial constructive work, GWW = Global myocardial wasted work, GWE = global myocardial work efficiency. BSA = body surface arena, HR = heart rate, SAP = Systolic arterial pressure, DAP = diastolic arterial pressure, LVEF = left ventricular ejection fraction, LV GLS = left ventricle global longitudinal strain.

Supplementary Table S2. Inter- and intra-observer reliability analysis.

Measurements	*ICC Inter-Observer*	*ICC Intra-Observer*	*CV Inter-Observer*	*CV Intra-Observer*
GWI (mmHg%)	0.914 (0.793–0.947)	0.814 (0.781–0.937)	9.7	7.5
GCW (mmHg%)	0.77 (0.628–0.819)	0.836 (0.648–0.889)	5.4	3.3
GWW (mmHg%)	0.91 (0.789–0.963)	0.932 (0.780–0.973)	10.7	7.7
GWE (mmHg%)	0.914 (0.798–0.947)	0.737 (0.638–0.851)	5.5	3.4

CV, coefficient of variation; GMW, global myocardial work; GCW, global myocardial constructive work; GWW, global myocardial wasted work; GWE, global myocardial work efficiency; ICC, intraclass correlation coefficient.
